# Health Tract, No. 157

**Published:** 1865

**Authors:** 


### HEALTH TRACT, No. 157.

### ESCAPING FROM FIRE.

Human life has been often thrown away from persons not taking the precaution to accustom their minds to dwell at times on the proper method of acting in emergencies; from want of this, many rush into the very jaws of death, when a single moment's calm reflection would have pointed out a certain and easy means of escape. It is the more necessary to fix in the mind a general course of action in case of being in a house while it is on fire, since the most dangerous conflagrations occur at dead of night, and at the moment of being aroused from a sound sleep the brain is apt to become too confused to direct the bodily movements with any kind of appropriateness, without some previous preparation in the manner contained herein. The London Fire Department suggests, in case the premises are on fire, to,

1. Be careful to acquaint yourself with the best means of exit from the house, both at the top and bottom.

2. On the first alarm, reflect before you act. If in bed at the time, wrap yourself in a blanket or bedside carpet. Open no more doors than are absolutely necessary, and shut every door after you.

3. There is always from eight to twelve inches of pure air close to the ground; if you can not, therefore, walk upright through the smoke, drop on your hands and knees, and thus progress. A wetted silk handkerchief, a piece of flannel, or a worsted stocking, drawn over the face, permits breathing, and, to a great extent, excludes the smoke.

4. If you can neither make your way upward nor downward, get into a front room; if there is a family, see that they are all collected here, and keep the door closed as much as possible, for remember that smoke always follows a draught, and fire always rushes after smoke.

5. On no account throw yourself, or allow others to throw themselves, from the window. If no assistance is at hand, and you are in extremity, tie the sheets together, having fastened one side to some heavy piece of furniture, and let down the women and children one by one, by tying the end of the line of sheets around the waist, and lowering them through the window that is over the door, rather than one that is over the area. You can easily let yourself down after the helpless are saved.

6. If a woman's clothes catch fire, let her instantly roll herself over and over on the ground. If a man be present, let him throw her down and do the like, and then wrap her up in a rug, coat, or the first *woolen* thing that is at hand.

Of the preceding suggestions, there are two which can not be too deeply engraven on the mind, that the air is comparatively pure within a foot of the floor, and that any wetted silk or woolen texture thrown over the face excludes smoke to a great extent; it is often the case that the sleeper is awakened by the suffocating effects of the smoke, and the very first effort should be to get rid of it, so as to give time to compose the mind, and make some muscular effort to escape.

In case any portion of the body is burned it can not be too strongly impressed on the mind that putting the burned part under water, or milk, or other bland fluid, gives instantaneous and perfect relief from all pain whatever; and there it should remain until the burn can be covered perfectly with half an inch or more of common wheaten flour, put on with a dredging-box, or in any other way, and allowed to remain until a cure is effected, when the dry, caked flour will fall off, or can be softened with water, disclosing a beautiful, new, and healthful skin, in all cases where the burns have been superficial. But in any case of burn, the first effort should be to compose the mind, by instantaneously removing bodily pain, which is done as above named; the philosophy of it being, that the fluid, whether water, milk, oil, etc., excludes the air from the wound; the flour does the same thing; and it is rare indeed that water and flour are not instantaneously had in all habitable localities.

